# Liver-specific *NG37* overexpression leads to diet-dependent fatty liver disease accompanied by cardiac dysfunction

**DOI:** 10.1186/s12263-016-0529-z

**Published:** 2016-05-21

**Authors:** Xin Zhou, MengMeng Xu, Liyang Wang, Yulian Mu, Rui Feng, Zhilong Dong, Yuexin Pan, Xunzhang Chen, Yongfeng Liu, Shangen Zheng, Donald D. Anthony, Jianjie Ma, Williams B. Isaacs, Xuehong Xu

**Affiliations:** 1 College of Life Sciences, Shaanxi Normal University, Xi’an, Shaanxi 710062 China; 2 Department of Pharmacology, Duke University Medical Center, Durham, NC 27708 USA; 3 State Key Laboratory for Animal Nutrition, Institute of Animal Science, Chinese Academy of Agricultural Sciences, Beijing, 100193 China; 4 Lanzhou University School of Medicine, Lanzhou, 730030 China; 5 Case Western Reserve University School of Medicine, Cleveland, OH 44106 USA; 6 Wuhan General Hospital of Guangzhou Military Command, Wuhan, Hubei 430070 China; 7 College of Food Engineering and Nutritional Science, Shaanxi Normal University, Xi’an, Shaanxi 710062 China; 8 Ohio State University School of Medicine, Columbus, OH 43210 USA; 9 Johns Hopkins School of Medicine, Baltimore, MD 21287 USA

**Keywords:** Cardiac dysfunction, Diet-dependent, Liver enlargement, NG37

## Abstract

**Background:**

Environmental factors are well-known causes of diseases. However, aside from a handful of risk indicators, genes’ encoding susceptibility to chronic illnesses and their associated environmental triggers are largely unknown. In this era of increasingly rich diets, such genetic predispositions would be immensely helpful from a public health perspective. The novel transgenic mouse model with liver-specific *NG37* overexpression characterized in this article identifies the diet-dependent function of *NG37* in the pathogenesis of fatty liver disease and cardiac arrhythmia.

**Results:**

The liver-specific *NG37* overexpression transgenic mouse model described here was generated using the Alb-SV40 polyA expression plasmid backbone. *NG37* cDNA under control of the albumin promoter for liver-specific expression was fused with a 5′ terminal M2 FLAG sequence and a SV40 early region transcription terminator/polyadenylation site attached at the 3′-UTR. These *NG37* transgenic mice developed normally and were physiologically normal on a standard diet. However, in comparison to non-transgenic (nTG) litter mates, these mice develop dramatic phenotypes within 12–18 days of starting a high-fat diet: (i) increased body weight (28.5 ± 12.3 g), (ii) increased liver weight (87.4 ± 35.7 mg), (iii) increased heart weight (140 ± 38.4 mg), and (iv) cardiac arrhythmia. The enlarged livers of high-fat diet *NG37* transgenic mice was histologically similar to human fatty liver disease and contained Maltese cross birefringent active depositions in hepatocytes that are indicative of fatty liver disease. We also confirmed via X-ray diffraction the steatotic vesicles in the diseased hepatocytes of our high-fat diet *NG37* mice was composed of cholesteryl derivatives also found in human fatty liver disease. In addition to cardiac enlargement, *NG37* transgenic mice on high-fat diet also exhibited highly irregular bradycardia not present in either high-fat diet nTG littermates or normal-diet transgenic litter mates.

**Conclusions:**

The dramatic high-fat diet-dependent symptoms (increased body weight, cardiac enlargement, fatty liver, and cardiac arrhythmias) characterized in our liver-specific *NG37* overexpression mouse model identifies *NG37* as a gene encoding latent lipid metabolism pathology induced only in the presence of an environmental factor relevant to human health: high-fat diet.

## Background

Diet is considered a causative factor in many human diseases such as cardiac disease, diabetes, and hypertension. We also know that genetics plays a significant role in determining susceptibility to these chronic illnesses [1, 2]. However, two individuals with the same environmental risk factors (over-rich diet, sedentary lifestyle, etc.) may not both develop diabetes. While this difference in susceptibility to the disease is thought to be genetic, we do not yet know the specific genes responsible for this phenotypic difference. In this study, we report the ability of a high-fat diet to induce fatty liver disease and cardiac arrhythmia in mice with liver-specific overexpression of the *NG37* gene.

*NG37* encodes the NG37 protein, which is a member of the von Willebrand A (vWA) superfamily. Proteins from the vWA superfamily all contain a vWA domain and participate in a diverse range of cellular functions including transcription, DNA repair, and ribosomal/membrane transport [3–6]. *Him*-*4*, the first vWA gene discovered via genetic analysis of *Caenorhabditis elegans*, encodes a vital extracellular matrix protein [3, 7, 8]. In mammals, this gene has two orthologs: fibulin-6/hemicentin-1 (*F6*/*H1*) and fibulin-8/hemicentin-2 (*F8*/*H2*), which encode fibulin-6/hemicentin-1 and fibulin-8/hemicentin-2, respectively. These two proteins share the vWA domain at their amino terminus with NG37. Recent studies have proven that these hemicentin proteins are required for successful mitosis during embryonic development [9–12]. However, despite the high homology of NG37 amino terminal region with these two previously characterized proteins, the actual function of NG37 is still unknown.

Our previous screening data have shown that *NG37* is highly expressed in the liver at the RNA level, indicating that this gene could play a critical role in this vital organ. Thus, we chose to explore the function of NG37 in the liver by generating mice with *NG37* overexpression driven by the liver-specific albumin promoter. Although these transgenic (TG) mice developed normally on a standard diet, when exposed to a high-fat diet, they rapidly developed pathologies. The most readily appreciated pathology was liver enlargement with hepatic lipid accumulation as identified by Maltese cross optical birefringence activity. This hepatosteatosis was confirmed by X-ray diffraction to be composed of the cholesterol accumulates also found in human fatty liver disease. In addition to fatty liver, these TG animals also developed severe cardiac arrhythmias likely as sequelae to disrupted lipid homeostasis. Our data suggest that overexpression of *NG37* predisposes the mice to fatty hepatic enlargement and cardiac dysfunction when triggered by a fat-enriched diet. These findings identify a direct, previously unknown link between the intertwined roles of environmental factors and genetic predisposition in pathogenesis: the ability of high-fat diet to unmask a susceptibility to fatty liver disease encoded by the *NG37* gene.

## Results and discussion

### Generation of the liver-specific Alb-*NG37*-overexpressing mouse model

**Fig. 1:**
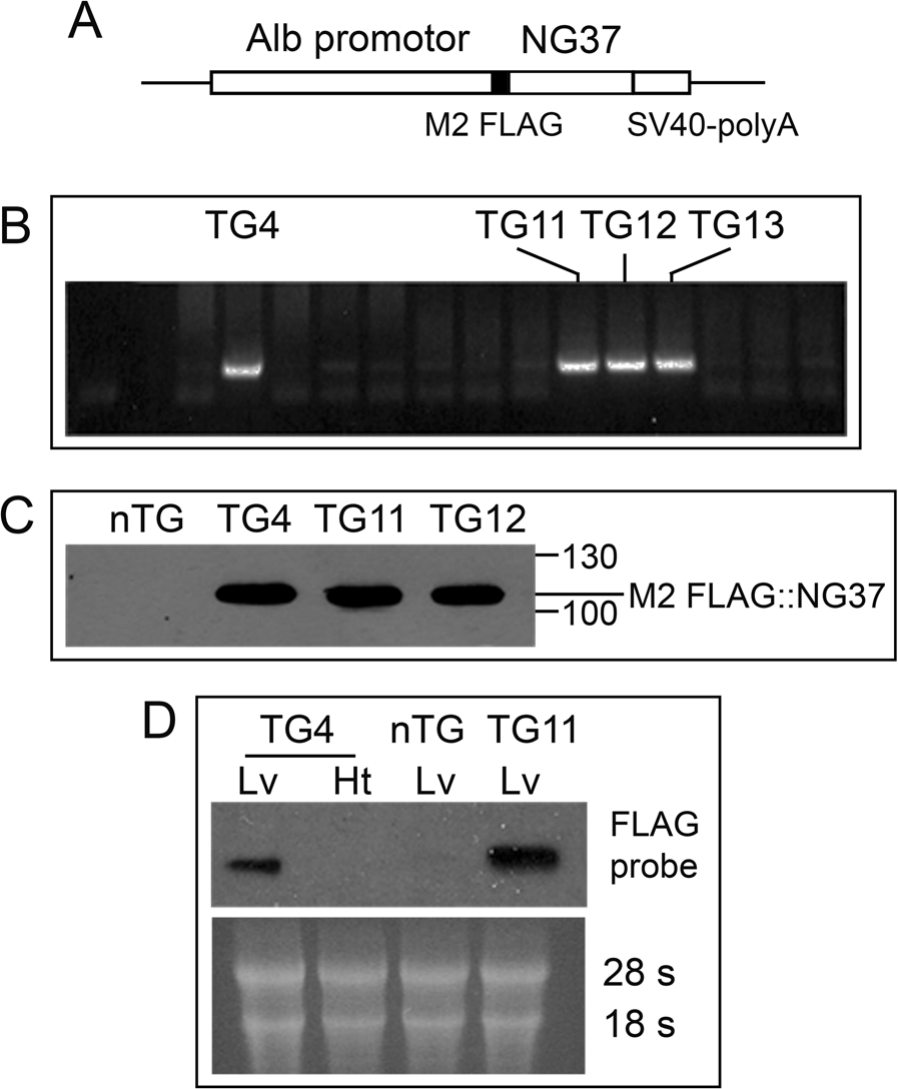
Generation of liver-specific Alb-*NG37* transgenic mice. **a** M2 FLAG::*NG37* fusion protein was subcloned into the Alb promoter-driven expression vector with SV40-poly A attached downstream of the minigene. **b** PCR was used to screen for positive transgenic (TG) mice. **c** Monoclonal antibody against M2-FLAG confirmed the expression of *NG37* in TG livers. **d** Northern blot confirmed the expression of *NG37* mRNA in TG livers (TG4 and TG11), but not in the hearts of transgenic mice (*second lane from left*) or non-transgenic (nTG) wild-type liver (*third lane*)

As will be described in “Methods” section, the Alb-*NG37* transgene construct (Fig. 1a) was injected into the pronucleus of zygotes collected from E0.5 day pregnant females. After brief culture in M16, zygotes were transferred into pseudopregnant females. After a full gestational term and natural birth, tail clippings from newborn pups were screened for the transgene by PCR (Fig. 1b).

To validate the liver-specific expression of *NG37*, liver samples were collected from Alb-*NG37* transgenic mice and subsequently lysed for protein extraction. Immunoblotting with the M2-FLAG monoclonal antibody was used to confirm *NG37* expression in the liver (Fig. 1c). Secondary confirmation with northern blot using a NG37 cDNA probe (M2-FLAG cDNA) confirmed that the minigene was only expressed in the liver and not in the heart of transgenic mice. We also confirmed that the minigene was not expressed in non-transgenic (nTG) littermate controls (Fig. 1d).

### Alb-*NG37* TG mice developed normally and were indistinguishable from non-transgenic littermates

**Fig. 2:**
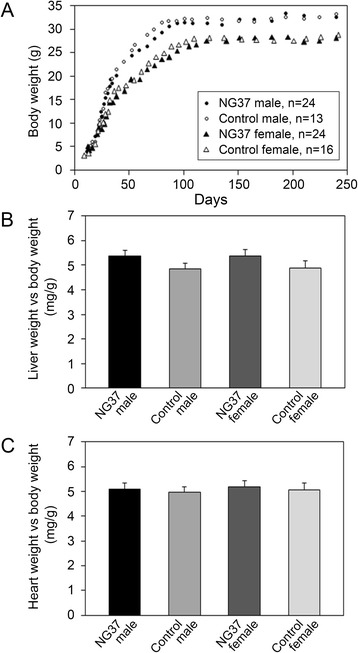
Comparison of growth curve, liver/body weight ratio, and heart/body weight ratio of Alb-*NG37* TG mice and nTG littermate controls. **a** Growth curve of the TG (male, *n* = 24; female, *n* = 24) and nTG (male, *n* = 13; female, *n* = 16) littermate control from birth to 8 months. **b** No biologically or statistically significant difference was seen between the liver weight/body weight ratios of male (TG *n* = 24, nTG *n* = 13; *p* > 0.05) or female (TG *n* = 24, nTG *n* = 16; *p* > 0.05) *NG37* and nTG littermates. **c** No biologically or statistically significant difference was seen between the heart weight/body weight ratios of male (TG *n* = 24, nTG *n* = 13; *p* > 0.05) and female (TG *n* = 24, nTG *n* = 16; *p* > 0.05) *NG37* and nTG littermates. All analysis was performed on mice reared on a normal diet

During phenotypic characterization of the Alb-*NG37* TG mice, all mice were maintained on a normal diet and their growth curves, liver/body weight ratios, and heart/body weight ratios were documented. Although TG mice had a slightly slower growth curve in comparison to their nTG littermate controls, mice achieved the same adult weight and the differences in growth curves were not statistically significant. This observation held true for both male (TG, *n* = 24; nTG *n* = 13) and female mice (TG, *n* = 24; nTG, *n* = 16) (Fig. 2a). There was also a slight and statistically insignificant increase in the liver/body weight ratio (male TG 5.11 ± 1.81; male nTG 4.92 ± 1.71; female TG 5.08 ± 1.24; female nTG 4.93 ± 1.62) and heart/body weight ratio (male TG 4.90 ± 1.70; male nTG 4.89 ± 1.32; female TG 4.96 ± 1.26; female nTG 4.95 ± 1.62) of Alb-*NG37* TG mice in comparison to nTG littermates at 4 months (Fig. 2b, c).

### High-fat diet-triggered liver enlargement with liquid crystal lipid droplet accumulation in Alb-*NG37* animals

**Fig. 3:**
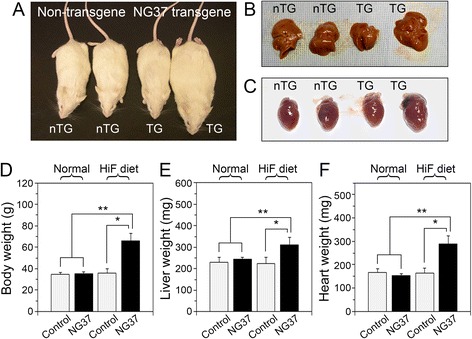
HiF diet Alb-*NG37* mice developed dramatic phenotypes in comparison to HiF diet nTG, normal-diet Alb-*NG37*, and normal-diet nTG littermates. **a** Gross differences in body size are observed in HiF diet Alb-*NG37* mice (TG#2) in comparison to normal-diet Alb-*NG37* (TG#1), normal-diet nTG (nTG#1), and HiF diet nTG littermates (nTG#2). Weight increase quantified in panel **d** notes that body weight of HiF diet TG mice (*n* = 18) significantly exceeded that of normal-diet TG and nTG mice (*n* = 18 each, *p* < 0.01) and HiF diet nTG mice (*n* = 16, *p* < 0.05). HiF diet Alb-*NG37* mice also exhibited liver enlargement **(b)** and cardiac enlargement **(c)** quantified as organ weight relative to body weight (**e**, **f**, respectively). **e** Liver weight of HiF diet TG mice (*n* = 18) significantly exceeded that of normal-diet TG and nTG mice (*n* = 18 each, *p* < 0.01) and HiF diet nTG mice (*n* = 16, *p* < 0.05). **f** Heart weight of HiF diet TG mice (*n* = 18) significantly exceeded that of normal-diet TG and nTG mice (*n* = 18 each, *p* < 0.01) and HiF diet nTG mice (in **e**, *n* = 16, *p* < 0.05). **p < 0.01; *p < 0.05

As previously discussed, Alb-*NG37* mice fed a normal diet were phenotypically indistinguishable from nTG litter mates. However, a dramatic phenotype develops in *NG37* transgenic animals when adult mice were switched to a high-fat (HiF) diet composed of the standard diet fried in a solution of 5 % cholesterol homogenized in corn oil. Here, we note that while creating our HiF diet directly from regular chow may produce batch effects and slightly different conditions from the industry standard high-fat diets, the deep frying technique we used reflects the increase of deep fried foods in the human diet. Within 12–18 days of diet change, Alb-*NG37* mice fed a HiF diet displayed a significant increase (*p* < 0.05) in body weight, liver/body weight ratio, and heart/body weight ratio. Body weights of HiF diet Alb-*NG37* mice doubled in comparison to normal-diet Alb-*NG37* and nTG littermates. More importantly, this dramatic phenotype remained just as true in a comparison between HiF diet Alb-*NG37* mice and HiF diet nTG litter mates (Fig. 3a–c). Specifically, in comparison to normal-diet Alb-*NG37* mice (*n* = 18), normal-diet nTG mice (*n* = 16), and HiF diet nTG mice (*n* = 16), HiF diet Alb-*NG37* mice (*n* = 18) demonstrated a statistically significant (*p* < 0.05) gain of 28.5 ± 12.3 g in body weight, 87.4 ± 35.7 mg in liver weight, and a doubling of heart weight at 140 ± 38.4 mg (Fig. 3d–f).

**Fig. 4:**
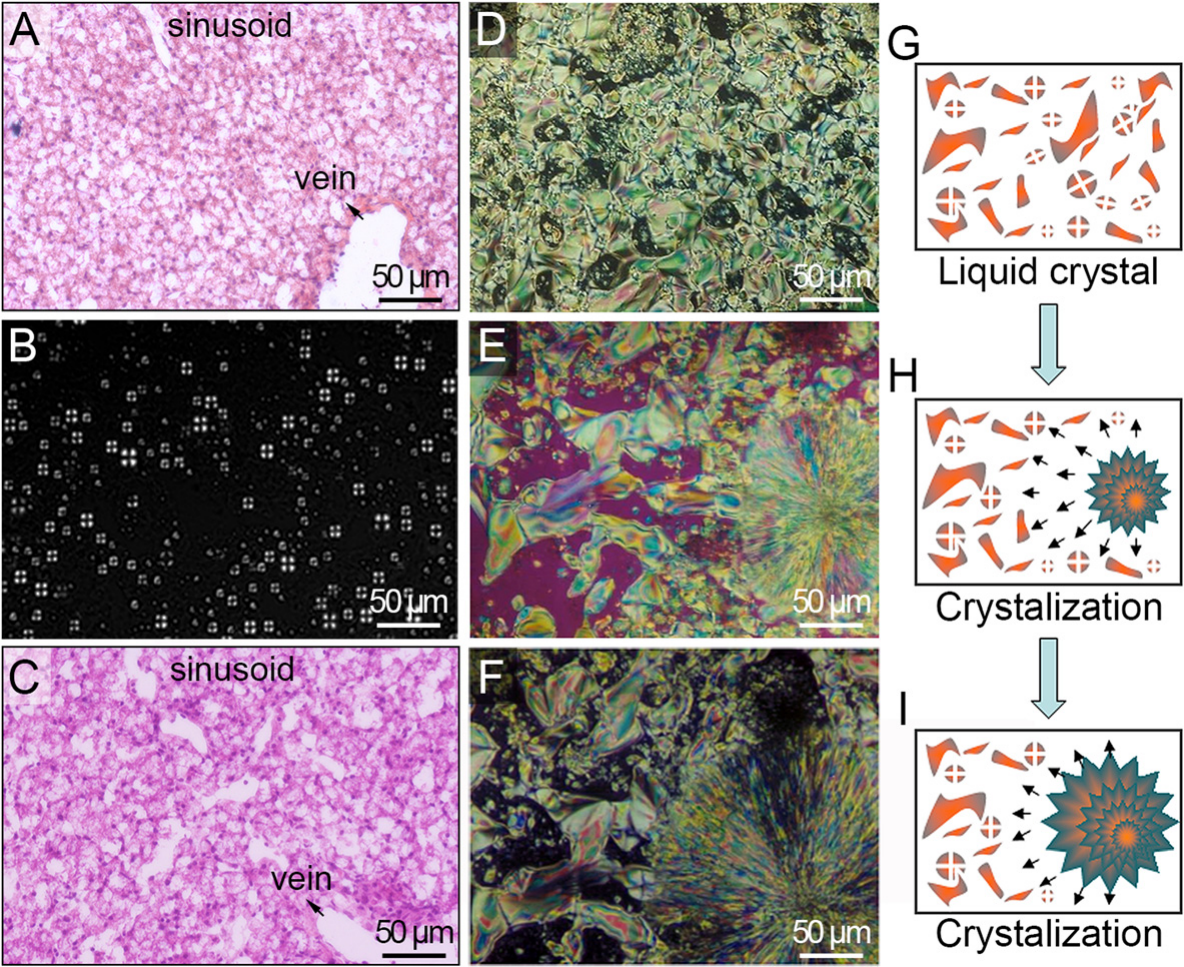
Histological and phase-transitional characterization of enlarged livers from HiF diet Alb-*NG37* mice. **a** Enlarged livers of HiF diet Alb-*NG37* mice exhibited increased steatotic vesicles in hepatocytes in comparison to **c** the livers of normal-diet Alb-*NG37*mice. **b** The livers of HiF diet Alb-*NG37* mice demonstrate Maltese cross activity. The transition from liquid crystal phase (**e**) to crystalline phase (**d**–**f**) was documented on samples extracted from enlarged Alb-*NG37* livers. **g**–**i** A schematic of the crystallization process from an initiating “seed” crystal is presented in step to the photographed process in panels **d**–**f**. *Scale bars* are 50 μm in length

To characterize the enlarged livers, we assessed the histology and phase-transitional properties of liver sections from HiF diet Alb-*NG37* mice and their HiF diet nTG littermates using a previously described protocol for animal development [13–17]. While the livers of HiF diet Alb-*NG37* mice retained normal liver structure on hematoxylin and eosin staining, the hepatocytes were enlarged and reticulated with large and small aggregates of steatosis (Fig. 4a) not seen in the livers of HiF diet nTG littermates (Fig. 4c), indicating that HiF diet Alb-*NG37* mice suffered from fatty liver disease. This was confirmed by the fact that HiF diet Alb-*NG37* hepatocytes contained liquid crystal Maltese crosses (Fig. 4b) while hepatocytes of HiF diet nTG mice did not (not shown). In humans, steatotic vesicles are composed of triglycerides and known to exhibit liquid crystal properties. Thus, we performed phase transition and X-ray diffraction studies on the liquid crystals we isolated from HiF diet Alb-*NG37* mouse livers to confirm the chemical composition of the hepatosteatosis.

As expected, temperature-catalyzed phase-transition studies conducted on Maltese cross birefringent active HiF diet Alb-*NG37* livers maintained a liquid crystalline form and exhibited changes in birefringent texture typical of liquid crystals (Fig. 4d–f). Consistent with triglyceride behavior in previous developmental studies of cholesteryl, these liquid crystals also demonstrated the rapid transition from liquid crystal to full crystallization via nucleation from an initial “seed” crystal (schematic in Fig. 4g–i). These Maltese cross optical birefringent activities were morphologically similar to those found in hepatic liquid crystal lipid droplets (LCLDs) during chicken embryo development, indicating a reactivation of developmental pathways in fatty liver disease [13–19].

**Fig. 5:**
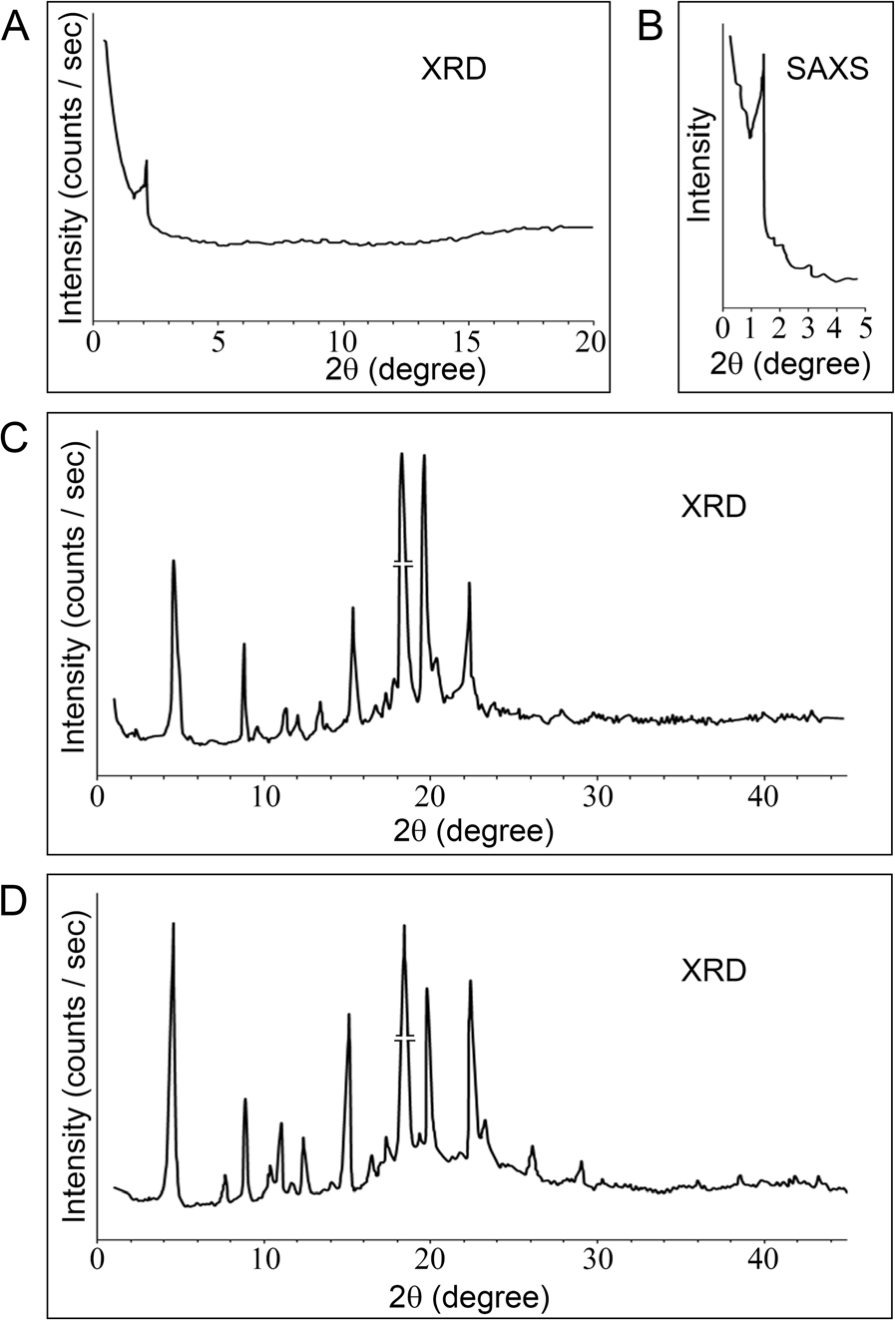
XRD and SAXS characterization of extracts from steatotic vesicles of HiF diet Alb-*NG37* mouse hepatocytes. Diffraction patterns of the extracts from HiF diet Alb-*NG37* mice (*n* = 6) in liquid crystalline phase had a single diffraction peak by XRD diffraction angle (2θ) of 0.6–20° (**a**) and SAXS angle (2θ) of 0.3–5° (**b**). The XRD pattern of the same extracts in crystalline phase displayed a patterned set of diffraction peaks within XRD diffraction angle (2θ) of 0.6–45° (*n* = 4) (**c**), which is comparable to the standard XRD pattern of cholesteryl oleate (**d**)

To confirm that the steatosis in our HiF diet Alb-*NG37* is pathologically similar to human fatty liver disease, we determined the chemical composition of the steatotic vesicles via X-ray diffraction (XRD) and small-angle X-ray scattering (SAXS). The content of the steatotic vesicles in liquid crystal phase displayed a single X-ray scattering peak at 2.44° within the XRD 2θ detection spectrum of 0.6° to 20° and was confirmed with SAXS within detection spectrum of 0.3 to 20° (Fig. 5a, b). By Bragg’s equation, the 2.44° value indicates a 36.8 Å lattice plane distance (dÅ). The XRD diffraction analysis of the sample in its fully crystalline form identified diffractions at lattice planes *d*(Å) of 19.31, 17.06, 5.90, 4.90, 4.59, and 4.09 corresponding to *I*/*I*_0_ (100), *I*/*I*_0_ (12), *I*/*I*_0_ (10), *I*/*I*_0_ (35), *I*/*I*_0_ (10), and *I*/*I*_0_ (8) in the detection spectrum of 0.6° to 45° (Fig. 5c). This XRD pattern matched the known diffraction of cholesteryl oleate (Fig. 5d and Table 1). Human fatty liver steatosis is also composed of cholesterol, indicating that hepatosteatosis in our HiF diet Alb-*NG37* mice shares the pathology of human fatty liver disease.

**Table 1:** X-ray diffraction pattern of the crystal transited from hepatic LCLDs in *NG37* transgenic mouse

Standard (cholesteryl oleate)		LCLC crystal, TG	
*I*/*I*_0_	*d*(Å)	*I*/*I*_0_	*d*(Å)
100	19.34	100	19.31
12	17.06	12	17.06
4	10.54	4	10.60
4	9.65	4	9.57
3	8.32	3	8.28
3	7.75	3	7.71
3	6.91	3	6.91
10	5.91	10	5.90
2	5.28	2	5.23
2	5.12	2	5.08
35	4.91	35	4.90
10	4.61	10	4.59
8	4.11	8	4.09

### HiF diet Alb-*NG37* TG mice exhibit cardiac arrhythmias

**Fig. 6:**
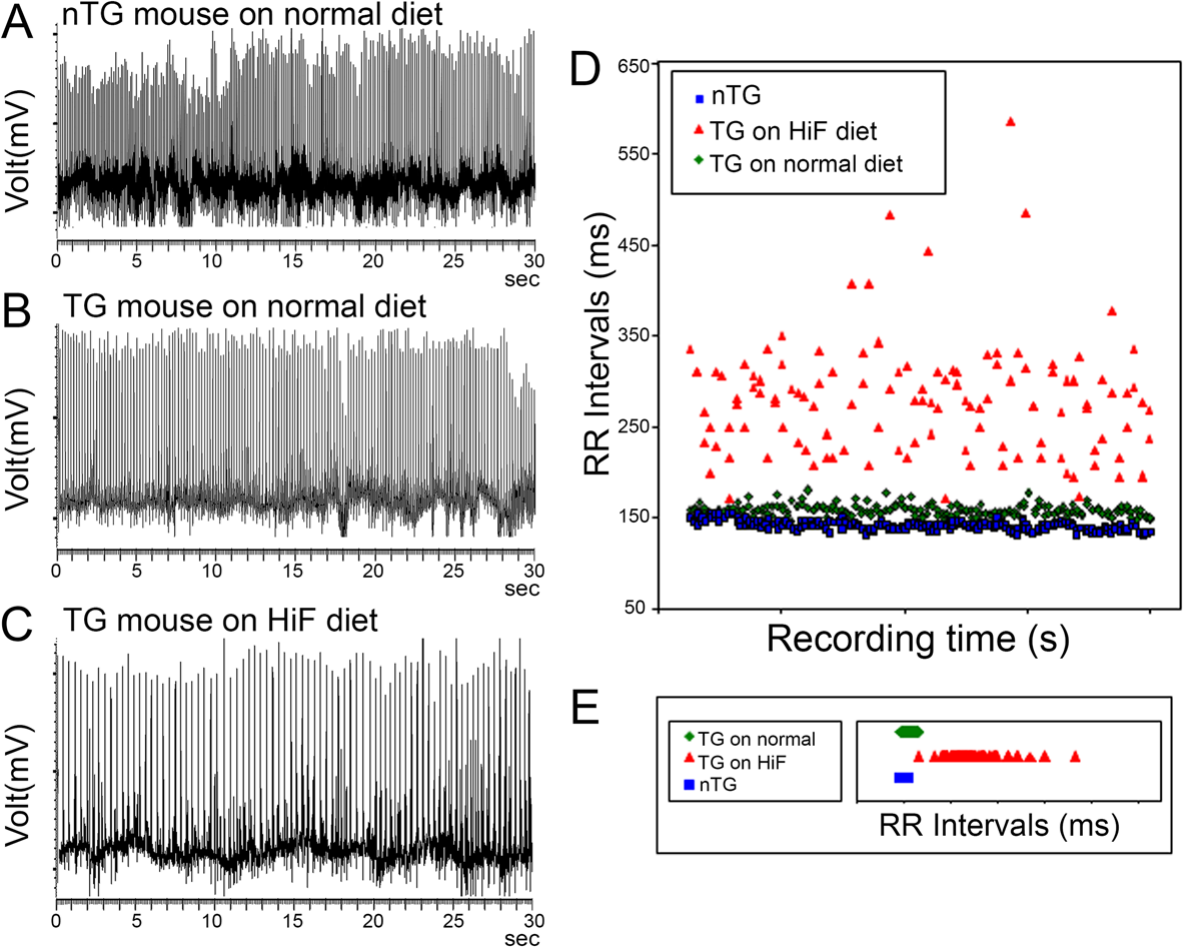
ECG and RR intervals of HiF diet Alb-*NG37* mice in comparison to normal-diet Alb-*NG37* mice and normal-diet nTG littermates. **a** Normal-diet nTG littermates have EC tracings and heart rates comparable to **b** ECGs of normal-diet Alb-*NG37* mice. **c** HiF diet Alb-*NG37* mice have a significantly decreased heart rate noticeable on ECG tracing. **d**, **e** The RR interval of 160 ± 35 ms in normal-diet Alb-*NG37* mice (*green diamond*) is consistent over time and comparable to the 150 ± 10 ms in normal-diet nTG mice (*blue square*) whereas HiF diet Alb-*NG37* TG mice (*red triangle*) have RR intervals of 260 ± 85 ms that range from 160 to 580 ms over time with 95 % of RR intervals distributed between 200 and 330 ms

Cardiac enlargement is a common symptom of many heart diseases. In order to better understand the physiological effects of doubled heart/body weight ratio in HiF diet Alb-*NG37* transgenic mice, we performed electrocardiogram (ECG) on HiF diet Alb-*NG37* mice. Prior to initiation of the HiF diet, Alb-*NG37* mice had a cardiac rate of 375 beats per min (bpm) and RR interval of 160 ± 35 ms, which are comparable to the 400 bpm and RR intervals of 150 ± 10 ms seen in nTG littermate controls (Fig. 6a, b). However, Alb-*NG37* mice rapidly developed severe bradycardia after initiation of the HiF diet (Fig. 6c). Not only is the RR intervals in HiF diet Alb-*NG37* mice extremely prolonged, but the RR intervals also followed a much wider distribution than both normal-diet Alb-*NG37* mice and nTG littermates regardless of diet (Fig. 6d, e). HiF diet Alb-*NG37* mice had mean RR intervals of 260 ± 85 ms with 95 % of RR intervals distributed between 200 and 330 ms with the full distribution of RR intervals ranging dramatically from 160 to 580 ms (Fig. 6d, e). This diet-dependent 3.6-fold difference in RR intervals within a single mouse is highly indicative of an irregular arrhythmia, which suggests a pathologic over physiologic bradycardia.

Arrhythmia is a common occurrence of aging [20, 21]. Bradycardia has been reported in younger patients and may not require immediate intervention if symptomatically mild [22, 23]. However, at 4 months of age, our transgenic mice are considered young adults. This young age in addition to the highly irregular nature of the bradycardia seen in our model likely generates symptomatic events (light-headedness, blurry vision, etc.) we cannot measure in mice. As such, the irregular bradycardia seen in our mice cannot be considered a normal physiological event but rather a pathological event triggered by HiF diet. Since this pathology was only found in HiF diet mice with overexpression of *NG37* restricted to the liver, it is a reasonable conclusion that the fatty liver disease caused by the combination of HiF diet and *NG37* overexpression is the underlying cause of this arrhythmia. Liver disease pathology may have indirectly led to the arrhythmia by causing the dramatic body habitus change that can lead to hypertension-induced cardiac enlargement. More likely, the severe cholesterol buildup in the steatotic livers of Alb-*NG37* is a symptom of HiF-diet-triggered imbalanced lipid metabolism, which can also cause abnormal accumulation of arterial plaques. These plaques can also lead to electrical conduction disturbances caused by small infarctions damaging the atrioventricular node, sinoatrial node, or Purkinje fibers. A partial block with some successful conduction compensated by a slower escape pacemaker would explain the highly irregular bradycardic rhythm. While we have not yet observed sudden cardiac death resulting from bradycardia-induced oxygen deprivation or from major myocardial infarctions, these mice may be more susceptible to such pathologies as they age.

### The diet-associated function of *NG37* could be universal in mammals

The human protein encoded by *NG37* shares 86, 83, and 82 % identity with its bovine (*Bos Taurus*), porcine (*Sus scrofa*), and murine (*Mus musculus*) equivalents, respectively. The highly conserved nature of the *NG37* gene indicates that the function of this protein is crucial for mammalian life (Table 2). The function of the amino acid terminal vWA domain and carboxyl terminal MIDAS domain provides hints about the function of *NG37*; we still do not know the exact role of this gene. Although genotype/phenotype comparison and histocompatibility studies have associated the *NG37* gene to lung tumor susceptibility, no causative associations have been proven [4, 24, 25]. Our findings in this liver-specific *NG37* gain-of-function model elucidate a definitive function for *NG37* in dietary cholesterol processing. The data revealed a HiF-diet-triggered pathology (fatty liver, cardiac enlargement, and cardiac arrhythmias) in mice overexpressing *NG37* in the liver. This diet-associated phenotype argues that overexpression of *NG37* disrupts cholesterol metabolism, resulting in hepatosteatosis, increased body habitus, and cardiac disease.

**Table 2:**
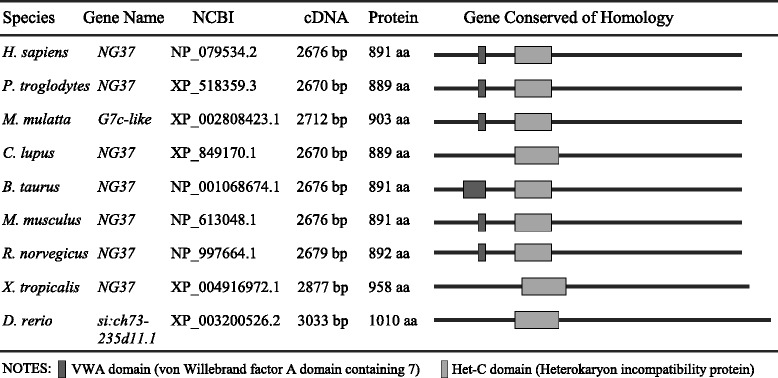
Schematic of the *NG37* gene and its homology

As a critical component of the cell membrane essential to maintaining the two-dimensional fluid membrane [26], cholesterol and its derivatives are often observed as massive birefringent Maltese crosses throughout the embryo and are found most abundantly in the liver [15, 17]. After embryonic development, birefringent Maltese crosses are only seen in pathology associated with erroneous reactivation of embryonic pathways. Amongst these diseases are age-related macular degeneration [27], steatohepatitis and atherosclerosis [28], Anderson-Fabry disease [14, 15], and cytoplasmic accumulation in macrophage-monocytes [14, 15]. The accumulation of liquid crystals in steatohepatitis was first observed in patients and later characterized in an animal model [28, 29]. Our data show that overexpression of *NG37* in the presence of a HiF diet can also lead to liquid crystal accumulation in the liver pathologically similar to steatohepatitis. The reappearance of liquid crystal-forming cholesterol derivatives in the livers of our HiF diet *NG37* mice indicates the reactivation of normal embryonic developmental mechanisms in a pathological process similar to those seen in the other liquid crystal-related pathologies. As previously discussed, the steatotic vesicles in hepatocytes may also be a symptom of the same lipid metabolism imbalance that lead to arterial plaque accumulation and cardiac disease. The Maltese cross birefringent active steatotic droplets could be composed of LCLDs produced to buffer and protect the body from lipid and cholesterol accumulation. Thus, there are two alternative mechanisms behind this previously unknown association between *NG37* and fatty liver the disease: (1) a primary pathogenic process caused by *NG37* overexpression or (2) a pathogenic outcome from over activation of a protective mechanism.

## Conclusions

HiF diet triggers the formation of massive cholesterol-based liquid crystals in the hepatocytes of our liver-specific *NG37*-overexpressing mouse model. This pathology led to fatty liver disease that is not seen in HiF diet nTG littermates, indicating that *NG37* plays a role in cholesterol metabolism and homeostasis. When triggered, pathologic effects of *NG37* overexpression were not restricted to the liver alone. HiF diet Alb-*NG37* mice also suffered from an increased body habitus, a symptom expected in a mouse with disrupted lipid homeostasis. Interestingly, these mice also developed cardiac hypertrophy with heart/body weight ratios double than their HiF diet littermate controls. This could be explained by cardiac compensation for increased body habitus-induced hypertension, a leading cause of pathologic cardiac hypertrophy. However, the speed with which this phenotype developed needs to be further studied as it could be due to hypercholesterolemia-induced vessel disease or neurohormonal changes. In fact, coronary artery infarct due to plaque buildup is the most likely cause of the irregular bradycardia we also characterized in this model. The wide RR interval distribution in these mice indicates sinoatrial node dysfunction or conduction failure as the most probable causes. Since we confirmed that our transgene is only expressed in the liver, damage to electrical transduction in the heart is most likely due to micro infarctions caused by diet-induced hypercholesterolemia in Alb-*NG37* mice. These findings indicate that overexpression of *NG37* denotes latent risk for fatty liver disease and its subsequent sequelae that is only uncovered in the presence of a HiF diet. With the increasing quality of life, rich diets that were previously unavailable to our ancestors have led to increasing public health concerns over expanding waistlines and metabolic diseases. However, few direct links between a specific gene and dietary habits have been described. Here, we describe a gene, *NG37*, overexpression of which could predispose a patient to early onset nonalcoholic fatty liver disease, increased body habitus, and cardiac disease when triggered by a HiF diet.

## Methods

### Animal maintenance and diet

DBA/-2J and C57BL/6J mice (The Jackson Laboratory; Bar Harbor, ME) and the genetically modified mice generated in this study were bred under standard conditions for maintenance. For sample collection, animals were euthanized by cervical dislocation prior to dissection. Tissues for RNA extraction were harvested immediately and snap frozen with liquid nitrogen, while tissues for histological analysis were fixed with 4 % paraformaldehyde in PBS immediately after collection. Samples for optical analysis were cryosectioned without delay. All animals were maintained in natural day-night cycle. All animal procedures were conducted under guidelines approved by the Animal Care and Use Committee of Xi’an for Animal Use of Universities in Shaanxi Province, P.R. China.

Experimental dietary alterations began when mice were 2 months of age. The control diet consisted of regular mouse chow (China National Standard GB14924.3-2010) purchased from Xi’an Fengwei Animals, Ltd. (Xi’an, China). HiF diet chow was produced in the following steps. First, a corn oil plus 5 % cholesterol (CCh; Sigma-Aldrich) solution homogenized by heating corn oil to boiling temperature then adding cholesterol and maintaining boiling temperatures for 15 min for full cholesterol dissolution. Regular mouse chow was then fried in the HiF solution for about 3 min to generate the fat-enriched foods. The fried HiF foods were considered ready to serve after cooling to room temperature. The HiF diet chow replaced normal chow in HiF diet groups. Both diets were provided on a free-taking principle.

### Cloning, construction of *NG37*, and model animal generation for liver-specific overexpression of *NG37*

Total RNA was extracted from mouse liver with Trizol (Life Technology) and evaluated by 0.7 % agarose electrophoresis for 28S RNA, 18S RNA, and 5.8S RNA. Reverse transcription polymerase chain reaction (RT-PCR) was employed to amplify the cDNA sequence of *NG37* from the total RNA. Poly(A)-RNA from total RNA was used to generate the first-strand cDNA with the cDNA synthesis kit according to the manufacturer’s instructions (Gibco BRL, Gaithersburg, MD). Based on the availability of the convenient endonuclease restriction sites, primer pairs (forward primer TTGCGGCCCTCCCTGTGGAGGTACCC and reverse primer CTGTCGACGGCTGCGTTGAGGGCCTC) were designed according to the published cDNA sequence for mouse *NG37* (ACCESSION: NM_138582 XM_994838). *Not* I and *Sal* I restriction sites were included in the forward primer and the backward primer at their 5′ ends, respectively, for ease of subcloning into pBlueScript II (+).

Due to lack of antibodies against *NG37*, a M2 FLAG (Asp-Tyr-Lys-Asp-Asp-Asp-Asp-Lys) was inserted before *NG37* in the reading frame within the expression construct. Monoclonal antibody of M2-FLAG would be used to distinguish the overexpression of the protein. The gene was subcloned under the control of the albumin promotor for liver-specific expression. SV40 early region transcription terminator and polyadenylation site were placed at the 3′-UTR. The primer sequences used to generate M2-FLAG::NG37 fusion cDNA were 5′TTGCGGCCGCCACCATGGATTACAAGGATGACGACGATAAGCTCCCTGTGGAGGTACCCCTGTCCCACCTG3′ and 5′CTGTCGACGGCTGCGTTGAGGGCCTCGCCCATACTG CC3′. Again, *Not* I and *Sal* I restriction sites on the 5′ of M2-FLAG and 3′ of *NG37* cDNA, respectively, were employed to insert the fusion cDNA into the Alb-SV40 polyA expression plasmid backbone. Highly purified Alb-*NG37* minigene made by CsCl purification was then prepared for pronuclei injection. The minigene was microinjected into C57BL/6J zygotes, after which the injected zygotes were implanted into the oviducts of pseudopregnant DBA/-2J. Alb-*NG37* transgenic mice were maintained in the DBA/-2J inbred strain.
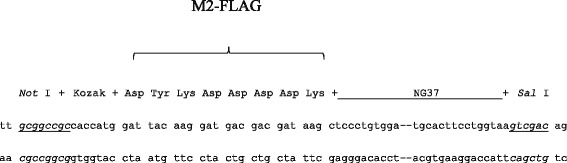


### RT-PCR, western blot, and northern blot

Total RNA was extracted from the mouse liver with Trizol (Life Technology). First-strand cDNA was obtained with the cDNA synthesis kit as described above. Protein samples for western blot analysis extracted from mice were separated on a 15 % Tris-glycine gel (Life Technology) and then transferred onto a polyvinylidene difluoride (PVDF) membrane. After blocking with 5 % non-fat milk in TBST, membranes were detected with a primary rabbit anti-flag antibody (Sigma-Aldrich) followed by a secondary goat anti-rabbit IgG conjugated with horseradish peroxidase (HRP) and developed after treatment with Supersignal West Pico Chemiluminescent Substrate (Pierce Biotechnology) as previously described [17].

For northern blot analyses, RNA extracted from tissue were run in 4 % formaldehyde agarose with formalin. Then, RNA were transferred onto nitrocellulose membrane and hybridized with H3-CTP-labeled M2-FLAG cDNA probe to visualize TG mRNA expression.

### Optical activity analyses and histology analyses

Cryosections were used for optical activity analyses. After dissection of mouse liver, the samples were set into cryomatrix embedding reagent (OCT) and placed in an aluminum foil basket. Samples were frozen by dipping the foil basket into liquid nitrogen. The frozen OCT block was then cut at thickness of 10–30 m. The sections were mounted with 20 % glycerol in PBS (pH 7.2) for polarization observation [14, 15, 17].

The samples obtained from the cryosection preparation were evaluated via two methods. First, conventional observation was carried out under non-crossed polarizer and analyzer to identify the distribution of birefringent activities. Then, birefringence in Maltese crosses of crystalline liquid crystal was documented. The observations were made on a XS-213A-P Polarizing Microscope (Jiangnan Jnoec Ltd., Nanjing).

Liquid crystal extracts were carried out according to previously published protocols [14, 15, 17]. Histological analysis was carried out on tissues fixed with 4 % neutral-buffered paraformaldehyde. The samples were processed for paraffin section and stained with hematoxylin and eosin (H&E) as previously published [13, 16].

### SAXS and XRD

For characterization of liquid crystalline samples, the content of steatotic vesicles were extracted from liver samples of HiF diet Alb-*NG37* mice and examined as previously described (Xu MM et al. 2012). SAXS patterns of the samples were obtained on the small-angle goniometer of D/max-rA diffractometer in diffraction/scattering angle (2θ) of 0.6–20° and 0.3–5° with CuKα radiation, Ni filter, 50 kV × 100–120 mA, and slit sizes 0.16-0.12-0.2-0.4 mm. Samples were clutched between two non-diffraction films to reduce movement and dehydration.

To further confirm the chemical composition as cholesterol derivatives, content of steatotic vesicles were extracted from liver samples of HiF diet Alb-*NG37* mice then crystallized following a previously described protocol [15, 17]. The XRD patterns of the crystal samples were conducted on the wide-angle goniometer of D/max-rA diffractometer in diffraction angle (2θ) of 0.6–45° with CuKα radiation, graphite monochromator, and slit sizes of 1/6°-1/6°-0.15 -0.45 mm. The results were then compared to the XRD pattern of cholesteryl oleate.

### Electrocardiographical analyses

Mice undergoing telemetric electrocardiographical measurements (Data Sciences International, St. Paul, MN) were anesthetized with 1.5 % isoflurane, and electrodes were surgically positioned in the intraperitoneal cavity following the lead II configuration according to manufacturer’s description [13]. Data were collected using an implantable radiofrequency micro-transmitter (TA1010ETA-F20) and recorded continuously using the waveform format hardware modification for multiple instrumented mice. The data analyses were performed using Dataquest A.R.T. version 4.1 (Data Sciences International).

### Ethics approval and consent to participate

All applicable international, national, and/or institutional guidelines for the care and use of animals were followed.

### Availability of data and material

The data set supporting the conclusions of this article are included within the article.

## Abbreviations

bpm: beats per min
ECG: electrocardiogram
F6/H1: fibulin-6/hemicentin-1
F8/H2: fibulin-8/hemicentin-2
H&E: hematoxylin and eosin
HiF: high-fat
HRP: horseradish peroxidase
LCLDs: liquid crystal lipid droplets
nTG: non-transgenic
OCT: cryomatrix embedding reagent
PVDF: polyvinylidene difluoride
RT-PCR: reverse transcription polymerase chain reaction
TG: transgenic
VWA: von Willebrand A
